# Growth Differentiation Factor 15 May Predict Mortality of Peripheral and Coronary Artery Diseases and Correlate with Their Risk Factors

**DOI:** 10.1155/2017/9398401

**Published:** 2017-07-17

**Authors:** Lung-An Hsu, Semon Wu, Jyh-Ming Jimmy Juang, Fu-Tien Chiang, Ming-Sheng Teng, Jeng-Feng Lin, Hsuan-Li Huang, Yu-Lin Ko

**Affiliations:** ^1^Cardiovascular Division, Department of Internal Medicine, Chang Gung Memorial Hospital and Chang Gung University College of Medicine, Taoyuan 33305, Taiwan; ^2^Department of Life Science, Chinese Culture University, Taipei 11114, Taiwan; ^3^Cardiovascular Center and Division of Cardiology, Department of Internal Medicine, National Taiwan University Hospital and National Taiwan University College of Medicine, Taipei 10002, Taiwan; ^4^Department of Research, Taipei Tzu Chi Hospital, Buddhist Tzu Chi Medical Foundation, New Taipei City 23142, Taiwan; ^5^Cardiovascular Center and Division of Cardiology, Department of Internal Medicine, Taipei Tzu Chi Hospital, Buddhist Tzu Chi Medical Foundation, New Taipei City 23142, Taiwan; ^6^School of Medicine, Tzu Chi University, Hualien 97071, Taiwan

## Abstract

Plasma GDF15 concentrations were measured in 612 Taiwanese individuals without overt systemic disease. Clinical parameters, *GDF15* genetic variants, and 22 biomarker levels were analyzed. We further enrolled 86 patients with PAD and 481 patients with CAD, who received endovascular intervention and coronary angiography, respectively, to examine the role of GDF15 level in predicting all-cause mortality. Significant associations were found between *GDF15* genotypes/haplotypes and GDF15 levels. The circulating GDF15 level was positively associated with age, smoking, hypertension, and diabetes mellitus as well as circulating levels of lipocalin 2 and various biomarkers of inflammation and oxidative stress. Kaplan-Meier survival analysis showed that baseline GDF15 levels of above 3096 pg/mL and 1123 pg/mL were strong predictors of death for patients with PAD and CAD, respectively (*P* = 0.011 and *P* < 0.001). GDF15 more accurately reclassified 17.3% and 29.2% of patients with PAD and CAD, respectively (*P* = 0.0046 and *P* = 0.0197), compared to C-reactive protein. Both genetic and nongenetic factors, including cardiometabolic and inflammatory markers and adipokines, were significantly associated with GDF15 level. A high level of GDF15 was significantly associated with an increase of all-cause mortality in patients with high-risk PAD and in patients with angiographically documented CAD.

## 1. Introduction

Growth differentiation factor 15 (GDF15) is a distinct member of the transforming growth factor-*β* cytokine superfamily with only a 15–29% similarity in amino acids to other members, suggesting that it plays a unique biological role [1]. Under healthy conditions, GDF15 is expressed at low levels in all organs, whereas it is expressed in high concentrations in the liver, kidney, heart and lungs in response to stress signals throughout adult life [2]. At the cellular level, GDF15 is produced and secreted by endothelial cells, macrophages, smooth muscle cells, and cardiac myocytes in response to ischemia, proinflammatory cytokine stimulation, oxidative stress, or mechanical stress [2]. Upon secretion, mature GDF15 rapidly diffuses into the circulation [3]. Circulating GDF15 levels have been used to predict disease progression in cancer, cardiovascular disease, chronic renal and heart failure, and pulmonary embolism [3–5]. GDF15 has also consistently been shown to be a strong predictor of cardiovascular, noncardiovascular, and all-cause mortality in healthy subjects and in those with diseases [5–10].

Previous studies have shown associations between genetic polymorphisms, clinical parameters and circulating levels of metabolic and inflammatory markers, and soluble GDF15 levels, although the results have been controversial [11–16]. GDF15 has also been reported to be expressed in adipose tissue and to be secreted as an adipokine by human adipocytes [17, 18]. In order to further investigate the possible mechanisms of GDF15 as a prognostic marker, we analyzed the associations of genetic determinants, clinical and biochemical markers including adipokines, and inflammatory markers with circulating GDF15 levels in a Taiwanese cohort. In addition, as the role of GDF15 levels and *GDF15* polymorphisms as a prognostic marker for peripheral artery disease (PAD) or coronary artery disease (CAD) has not previously been studied, we also enrolled patients with high-risk PAD and patients with CAD to evaluate the role of GDF15 levels and *GDF15* genetic polymorphisms in predicting long-term mortality.

## 2. Materials and Methods

### 2.1. Study Design and End Points

**Figure 1:**
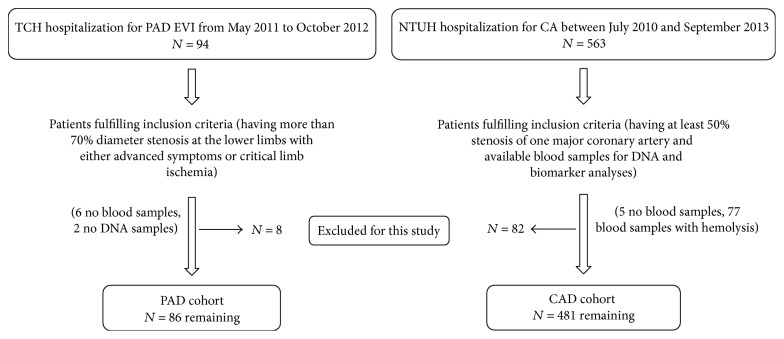
Study inclusion and exclusion criteria flow chart. This flow chart illustrates the inclusion and exclusion criteria used to screen peripheral artery disease (PAD) patients from Tzu Chi Hospital (TCH) and coronary artery disease (CAD) patients from National Taiwan University Hospital (NTUH), respectively. EVI: endovascular intervention; CA: coronary angiography.

The study was designed to elucidate the genetic and nongenetic correlates of GDF15 levels and their prognostic predictability of atherosclerotic cardiovascular disease in a Taiwanese population. A Taiwanese cohort from a cardiovascular health examination program was initially recruited to elucidate the genetic and biomarker determinants of conventional and emerging coronary risk factors, including GDF15. Two independent populations with atherosclerotic cardiovascular diseases were further prospectively enrolled, one with CAD patients and another with PAD patients, for the analysis of genetic and biomarker levels as long-term prognostic predictors in a Taiwanese population. A flow chart of patient enrollment with the inclusion and exclusion algorithm is shown in Figure 1. The primary end point of the latter two disease populations was all-cause mortality. This study was conducted in accordance with the Declaration of Helsinki principles.

### 2.2. Study Population

The control group was recruited during routine cardiovascular health examinations from October 2003 to September 2005 at Chang Gung Memorial Hospital and consisted of 612 Han Chinese subjects (323 men with a mean age of 45.6 ± 10.0 years and 289 women with a mean age of 47.0 ± 10.0 years) who responded to a questionnaire on their medical history and lifestyle characteristics. The clinical characteristics of the study population have been described elsewhere [19]. All of the participants provided written informed consent, and the study was approved by the Ethics Committee of Chang Gung Memorial Hospital and the Ethics Committee of Taipei Tzu Chi Hospital, Buddhist Tzu Chi Medical Foundation.

In the PAD group, 86 consecutive hospitalized patients with PAD who received endovascular intervention (EVI) and had no known history of malignancy were enrolled from May 2011 to October 2012 at Taipei Tzu Chi Hospital (Figure 1). The PAD lesions in these patients were all more than 70% diameter stenosis at the lower limbs with either advanced symptoms or critical limb ischemia (CLI) according to the recommended standards [20]. CLI of the lower extremities refers to a condition characterized by chronic ischemic at-rest pain, ulcers, or gangrene in one or both legs attributable to objectively proven PAD. All clinical data were obtained from the patients' medical records. The primary end point was all-cause mortality. All of the participants provided written informed consent, and the studies were approved by the Ethics Committee of Taipei Tzu Chi Hospital, Buddhist Tzu Chi Medical Foundation.

In the CAD group, a total of 481 patients with CAD who received coronary angiography (CA) and had at least 50% stenosis of one major coronary artery and who had available blood samples for DNA and biomarker analyses were recruited between July 2010 and September 2013 from National Taiwan University Hospital (Figure 1). All clinical data were obtained from the patients' medical records. The primary end point was all-cause mortality. Seven patients who were lost to follow-up after enrollment were contacted by a telephone before the end of the study. Three of these patients had died, and the cause of death was ascertained by the relatives. All of the participants provided written informed consent, and the study was approved by the Research Ethics Committee of National Taiwan University Hospital.

### 2.3. Genomic DNA Extraction and Genotyping

Genomic DNA was extracted as reported previously [19]. Seven single-nucleotide polymorphisms (SNPs) near and at the *GDF15* gene were chosen in this study (Supplementary Table S1 available online at https://doi.org/10.1155/2017/9398401). The tagSNPs rs8101804 and rs16982345 were selected by running the tagger program implemented in Haploview software. The rs749451, rs888663, rs1227731, and rs1054564 SNPs were selected because they were associated with the lowest *P* values in the genome-wide association study of GDF15 by Ho et al. [16]. The rs1058587 SNP was selected since previous studies have reported that it might be functional [21]. Genotyping for these SNPs was performed using TaqMan SNP Genotyping Assays obtained from Applied Biosystems (ABI, Foster City, CA, USA). For quality control purposes, approximately 10% of the samples were regenotyped in a blinded fashion and the same results were obtained.

### 2.4. Laboratory Examinations

A total of 15 mL of venous blood was collected in the morning after an overnight fast. Serum and plasma samples were obtained by centrifugation at 3000 ×g for 15 minutes at 4°C. Immediately after centrifugation, serum/plasma samples were frozen and stored at −80°C prior to analysis. Circulating plasma levels of GDF15, matrix metalloproteinase 1 (MMP1), soluble P-selectin (sP-selectin), and soluble tumor necrosis factor receptor II (sTNFRII) and serum levels of lipocalin 2 (LCN2), matrix metalloproteinase 2 (MMP2), and resistin were measured using commercially available ELISA kits (R&D, Minneapolis, MN, USA). Other markers, including serum C-reactive protein (CRP), serum amyloid A (SAA), homocysteine, soluble intercellular adhesion molecule (sICAM1), soluble vascular cell adhesion molecule (sVCAM1), soluble E-selectin (sE-selectin), adiponectin, leptin, matrix metalloproteinase 9 (MMP9), plasma monocyte chemotactic protein 1 (MCP1), urine creatinine, microalbuminuria, and 8-hydroxydeoxyguanosine (8-OHdG), were measured using a sandwich ELISA developed in-house. All in-house kits showed good correlation when compared with commercially available ELISA kits (supplemental references). Serum insulin levels were measured using an immunoradiometric assay (Biosource, Nivelles, Belgium). Overall, the intra- and interassay variability coefficients fell within the range of 1.8% to 9.5%. Mean intra-assay coefficients of variation (CVs) from plasma specimens were 1.8, 5.5, 3.0, 2.2, and 3.8% and interassay CVs were 5.6, 8.2, 8.8, 4.1, and 5.7% for GDF15, MMP1, sP-selectin, sTNFRII, and MCP-1 levels, respectively, while the intra-assay CVs from serum specimens were 3.6, 3.8, 7.7, 5.1, 7.1, 8.5, 2.1, 4.2, 4.1, 6.1, and 7.1% and interassay CVs were 5.6, 7.8, 7.0, 4.0, 9.5, 8.1, 3.8, 6.8, 3.4, 8.8, and 9.1% for LCN2, resistin, adiponectin, leptin, CRP, SAA, sICAM-1, sVCAM1, sE-selectin, MMP2, and MMP9 levels, respectively. Glucose levels were determined enzymatically using the hexokinase method, and total cholesterol (TCHO) and triglyceride levels were measured by automatic enzymatic colorimetry. High-density lipoprotein cholesterol (HDLC) levels were measured enzymatically after phosphotungsten/magnesium precipitation. Low-density lipoprotein cholesterol (LDLC) levels were either calculated using the Friedewald formula or, in patients with a triglyceride level > 400 mg/dL, measured using commercial reagents with a standard protocol. Plasma fibrinogen levels were determined using the Clauss method adapted for a Sysmex CA1-1500 instrument (Kobe, Japan). The homeostasis model assessment of insulin resistance (HOMA-IR) index was calculated by the formula: HOMA-IR = fasting serum insulin (*μ*U/mL) × fasting plasma glucose (mmol/L)/22.5. The estimated glomerular filtration rate (eGFR) was determined using the following equation: 194 × serum creatinine^−1.094^ × age^−0.287^ (×0.739 if female) [22]. Laboratory personnel performed ELISA and genotyping without knowledge of the clinical status of the subjects.

### 2.5. Definition of Baseline Measurements

Classic CAD risk factors including cigarette smoking, diabetes mellitus (DM), hypertension, hyperlipidemia, and obesity were defined as follows. Patients who smoked at least 1 cigarette per day at the time of the survey were regarded as smokers. DM was defined according to the guidelines of the American Diabetes Association as a fasting plasma glucose level ≥ 126 mg/dL, a glycohemoglobin value ≥ 6.5%, or having medical records documenting DM and receiving hypoglycemic agents. Hypertension was defined as a systolic blood pressure (BP) of ≥140 mmHg and/or a diastolic BP of ≥90 mmHg or having medical records documenting hypertension and receiving antihypertensive drugs. Obesity was defined as a body mass index (BMI) ≥ 25 kg/m^2^, according to the Asian criteria [23]. Metabolic syndrome was defined according to the ATP III Asian criteria.

### 2.6. Statistical Analysis

The clinical characteristics of the participants are expressed as means ± standard deviation (SD) and percentages, except when the distribution was strongly skewed, in which case the median and interquartile ranges are given. The chi-square test was used to examine the differences in categorical variables and to compare the allele and genotype frequencies. The continuous variables between groups were tested using a two-sample *t*-test or Mann–Whitney *U* test. All the biomarker levels were logarithmically transformed prior to statistical analysis in order to adhere to a normality assumption.

The genetic association study of GDF15 levels was conducted in the healthy population. The analysis of deviation from the Hardy-Weinberg equilibrium, estimation of linkage disequilibrium between polymorphisms, and association of SNP genotypes and SNP-derived haplotypes with log-transformed GDF15 level was performed using the Golden Helix SVS Win32 7.3.1 software (Golden Helix, Bozeman, MT, USA). The Hardy-Weinberg equilibrium (HWE) was assessed using Fisher's exact test. Measures of pairwise linkage disequilibrium (LD) and haplotype frequencies were estimated using the expected maximization algorithm (EM) in the HelixTree Genetics Analysis software (Golden Helix). A linear regression in Golden Helix was applied to capture the major effect of each polymorphism on the log-GDF15, with age, sex, BMI, and smoking as confounding covariates. These analyses were performed using additive, dominant, and recessive genetic models, respectively, for each SNP. In the haplotype association analysis, coefficients and *P* values for the selected haplotype, compared to all other unselected haplotypes, were estimated using haplotype trend regression analysis implemented in the HelixTree program. For the multiple comparisons, the false discovery rate (FDR) estimation method was applied to the genetic association analyses. Spearman partial correlation coefficients were further used to examine the relation of GDF15 and clinical and biochemical factors in the healthy population. A Bonferroni correction for multiple testing was used with *α* = 0.002 after the 22 different tested laboratory variables were taken into account. Univariate linear regression analysis was performed to analyze the association between GDF15 and classic CAD risk factors.

To evaluate GDF15 as a prognostic biomarker for PAD and CAD populations, a receiver operating characteristic (ROC) analysis was used to identify the sensitivity and specificity of GDF15 cutoff points for the prediction of all-cause mortality. The optimal cutoff values were defined as the point at which the value of “sensitivity + specificity − 1” was maximum (Youden's index). The survival curve was plotted with the Kaplan-Meier method, and the significance was examined by the log-rank test. The sample size was estimated by the formula described by Smith and Morrow [24]: the expected number of events (*e*) = *f* (*α*, *β*) × [(1 + RR)/(1 – RR)^2^], (RR as expected rate ratio, *f* = 7.85 at test size 5% and power 80%). Prior data indicate that annual mortality rates of the PAD and CAD populations were 5.7% and 3.0% per year [25]. Thus, to be able to detect a 10-fold increase in the mortality rate for patients with GDF15 levels above the threshold, with power of 80% at the 5% significant level, 35 person-years per arm are required. Net reclassification improvement (NRI) and integrated discrimination improvement (IDI), as described by Pencina et al. [26], were used to evaluate whether GDF15 improved mortality risk discrimination, as compared to CRP. The continuous NRI was computed using mortality rates of 1% and 5% to define low (<1%)-, intermediate (1–5%)-, and high (>5%)-risk categories. All calculations were performed with statistical SPSS (version 18 for Windows, Inc., Chicago, IL, USA) and SAS software (version 9.3, SAS Institute, Cary, NC, USA). Values of *P* < 0.05 using a two-sided test were considered statistically significant. Missing data were approached with listwise deletion.

## 3. Results

### 3.1. Clinical and Biochemical Characteristics

A summary of the demographic features, clinical profiles, and inflammatory biomarkers for the study participants is provided in Table 1. No significant deviations from the Hardy-Weinberg equilibrium were detected for the studied polymorphisms (*P* = 0.942, 0.979, 0.672, 0.719, 0.912, 0.736, and 0.967 for SNPs rs749451, rs888663, rs8101804, rs1227731, rs1058587, rs1054564, and rs16982345, resp.) (Supplementary Table S1). With the exception of rs749451, the other six SNPs were in strong pairwise linkage disequilibrium (LD) (Supplementary Figure S1).

### 3.2. Associations of the GDF15 Genotypes/Haplotypes with Circulating Levels of GDF15

Our results showed that genetic variants in or around the *GDF15* gene were significantly associated with GDF15 levels in our Taiwanese cohort (Table 2). After adjusting for age, sex, BMI, and smoking, significant associations with GDF15 level were observed for the three polymorphisms rs8101804, rs1227731, and rs1054564 using an additive inheritance model (FDR-adjusted *P* = 0.019, *P* = 4.87 × 10^−7^, and *P* = 4.87 × 10^−7^, resp.). With the dominant model, the minor alleles of rs8101804, rs1227731, and rs1054564 were found to be associated with a higher GDF15 level (FDR-adjusted *P* = 0.027, *P* = 7.59 × 10^−7^, and *P* = 7.59 × 10^−7^, resp.). With the recessive model, the minor alleles of rs1227731, rs1058587, and rs1054564 were found to be associated with a higher GDF15 level (FDR-adjusted *P* = 0.020, *P* = 0.020, and *P* = 0.020, resp.). In contrast, the minor allele of rs16982345 was associated with a lower GDF15 level in a recessive model (FDR-adjusted *P* = 0.020). Six common haplotypes (≥1% frequency) were derived from the seven SNPs, accounting for 97.89% of all inferred haplotypes. In haplotype analysis, one haplotype inferred from the seven SNPs (*CTTACCG*) was found to be associated with GDF15 level (FDR-adjusted *P* = 2.21 × 10^−6^) (Table 3).

### 3.3. Associations between GDF15 Levels and Clinical and Biochemical Correlates

The associations between GDF15 levels and clinical and biochemical factors are shown in Tables 4 and 5. After a Bonferroni correction for multiple testing, significant correlations were observed between GDF15 levels and age, as well as circulating levels of LCN2 and various inflammatory and oxidative stress markers, including sE-selectin, sVCAM1, sICAM1, sTNFRII, and homocysteine levels (Table 4). Further, we analyzed the associations between GDF15 levels and the presence or absence of several risk factors for cardiovascular disease. Plasma levels of GDF15 were significantly higher in those who currently smoked than in those who did not, in those with hypertension than in those without, in those with DM than in those without, in those with metabolic syndrome than in those without, and in men than in women after adjusting for age, sex, BMI, and smoking (*P* ≤ 0.001, *P* = 0.002, *P* < 0.001, *P* = 0.021, and *P* = 0.023, resp.) (Table 5). In contrast, no significant associations were noted between GDF15 levels and obesity or lipid traits.

### 3.4. GDF15 Levels, Polymorphisms, and Long-Term Mortality in the Patients with PAD

**Figure 2:**
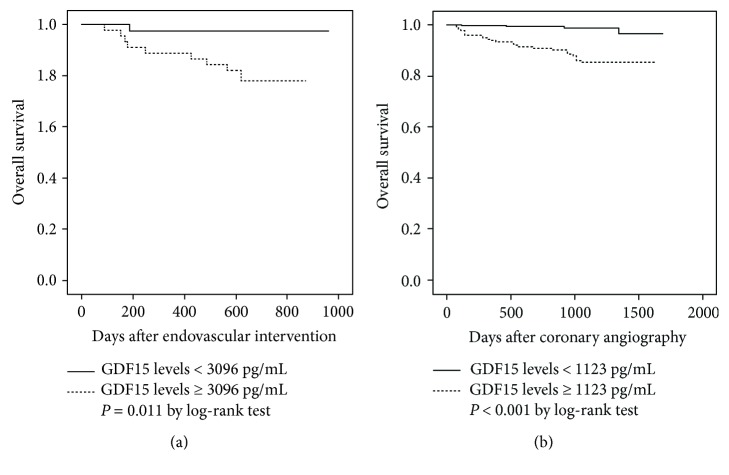
(a) Kaplan-Meier survival curves for patients with peripheral artery disease. Event-free survival curves for all-cause mortality at a mean follow-up of 21 months were plotted using the Kaplan-Meier method, and *P* values were calculated using the log-rank test. (b) Kaplan-Meier survival curves for patients with coronary artery disease. Event-free survival curves for all-cause mortality at a mean follow-up of 33 months were plotted using the Kaplan-Meier method, and *P* values were calculated using the log-rank test.

The PAD group consisted of 86 patients, and their baseline characteristics are shown in Table 6. GDF15 levels on the day of the EVI procedure ranged from 750.6 to 13788.3 pg/mL, with a median of 3448.7 pg/mL. Significantly higher GDF15 levels were noted in the patients with PAD compared to the controls (3448.7 (1713.5–5427.9) versus 535.0 (415.3–716.5) pg/mL, adjusted *P* < 0.001). The mean follow-up period (until the end of 2013) was 21 ± 6 months (range: 2 to 32 months), during which 10 patients died. Comparisons between the survivors and nonsurvivors are shown in Table 6. There were no significant differences between the two groups with regard to age, gender, smoking, BMI, hypertension, dyslipidemia, diabetes, end-stage renal disease, prior stroke, congestive heart failure, history of CAD, and circulating CRP levels. Notably, the patients who died had significantly higher baseline GDF15 levels compared to those who survived (5749.6 (3530.7–7072.0) versus 2849.4 (1669.5–5320.7) pg/ml, *P* = 0.028). Using ROC curve analysis and Youden's index, the best cutoff point for predicting the risk of mortality after EVI was a GDF15 level of 3096 pg/mL (90.0% sensitivity, 52.6% specificity). Kaplan-Meier survival analysis showed that a GDF15 level above 3096 pg/mL was a strong predictor of mortality (Figure 2(a); *P* = 0.011, statistical power = 0.495 at *α* = 0.05). Compared with CRP, GDF15 improved the predictive ability of mortality as shown by a continuous NRI of 0.17 (95% confidence interval, 0.06 to 0.29; *P* = 0.0046), in which the NRI for events was 0 and for nonevents 0.17. However, the IDI value was 0.02, which was not significant (*P* = 0.45). Although the patients with PAD and the rs1054564 C allele had higher baseline GDF15 levels (4407.8 (2362.3–7044.5) versus 2814.4 (1699.8–5417.2) pg/mL, *P* = 0.280) and higher probability (40% versus 28.9%) of mortality during follow-up after EVI compared to those with the rs1054564 GG allele, the differences were not statistically significant.

### 3.5. GDF15 Levels, Polymorphisms, and Long-Term Mortality in the Patients with CAD

The CAD group consisted of 481 patients, and their baseline characteristics are shown in Table 7. GDF15 levels on the day of CA ranged from 200.4 to 19524.2 pg/mL, with a median of 945.0 pg/mL. Significantly higher GDF15 levels were noted in the patients with CAD compared to the controls (945.0 (666.8–1552.4) versus 535.0 (415.3–716.5) pg/mL, adjusted *P* < 0.001). The mean follow-up period (until the end of 2014) was 33 ± 11 months (range: 5 to 1692 days), during which 27 patients died. Comparisons between the survivors and nonsurvivors are shown in Table 7. There were no significant differences between the two groups with regard to gender, smoking, BMI, hypertension, dyslipidemia, prior stroke history, and frequency of receiving coronary interventions. However, the mean age and CRP levels, frequencies of diabetes, prior congestive heart failure, and acute coronary syndrome on CA were significantly higher in the patients who died than in those who survived. Notably, patients who died had significantly higher baseline GDF15 levels compared to those who survived (2480.4 (1437.2–4278.7) versus 913.4 (656.4–1403.3) pg/ml, *P* < 0.001). Using ROC curve analysis and Youden's index, the best cutoff point for predicting the risk of mortality after CA was a GDF15 level of 1123 pg/mL (85.2% sensitivity, 65.4% specificity). Kaplan-Meier survival analysis showed that a GDF15 level above 1123 pg/mL was a strong predictor of mortality (Figure 2(b), *P* < 0.001, statistical power = 0.876 at *α* = 0.05). Compared with CRP, GDF15 improved the predictive ability of mortality as reflected by a continuous NRI of 0.29 (95% confidence interval, 0.06 to 0.52; *P* = 0.0197), in which NRI for events was 0.23 and for nonevents 0.06. However, the IDI value was 0.02, which was not significant (*P* = 0.51). Although the patients with CAD and the rs1054564 C allele had significantly higher baseline GDF15 levels (1021.6 (760.7–1885.5) versus 905.5 (633.2–1382.9) pg/mL, *P* = 0.009) compared with the rs1054564 GG carriers, the long-term mortality rates were not significantly different.

## 4. Discussion

In this study, we evaluated various genetic and nongenetic correlates of GDF15 levels in a Taiwanese cohort and a wide range of associations were noted. We confirmed that genetic polymorphisms around the *GDF15* gene were associated with GDF15 levels and also that GDF15 levels were associated with age, smoking, hypertension, and DM as well as circulating levels of LCN2 and many inflammatory and oxidative stress biomarkers including sTNFRII and homocysteine that have not been previously reported. In addition, high GDF15 levels rather than genetic polymorphisms were a strong and better predictor of mortality than CRP level in both patients with symptomatic PAD and those with angiographically documented CAD. These results suggest that GDF15 affects not only cardiac-related but also non-cardiac-related mortality.

### 4.1. Genetic Determinants of GDF15 Levels

Genetic variants around the *GDF15* gene locus have been shown to be associated with GDF15 concentration, accounting for up to 38% of the variability of GDF15 concentrations [16]. We found five SNPs (rs8101804, rs1058587, rs1227731, rs1054564, and rs16982345) in strong LD around the same chromosomal region, and the related haplotype was associated with GDF15 concentration in our Taiwanese cohort. In contrast to the findings in Caucasian populations, the SNPs rs1227731 and rs1054564 within GDF15 were the most significantly associated SNPs in our population, and both SNPs were found to be in complete LD. Both rs1227731 and rs16982345 (intronic SNP and 3′ downstream tagSNP, resp.) are probably nonfunctional, whereas rs8101804 has previously been shown to be a functional promoter SNP in strong LD with both rs1227731 and rs1054564 [21]. However, the association of rs1227731 with GDF15 levels was less significant than that of rs1054564 in our study. rs1054564 is located at the 3′-untranslated region of the *GDF15* gene and could potentially influence miR-1233 miRNA binding and thus GDF15 expression according to established miRNA target prediction programs such as MiRanda and TargetScan. miR-1233 has been also found to play a role in a plethora of diseases and is a potential marker for cancer and cardiovascular disease [27, 28]. Thus, our results suggest that rs1054564 may be the major genetic determinant of GDF15 concentration in a Taiwanese population.

### 4.2. Association with GDF15 Levels: Baseline Characteristics

All previous studies and our investigation have shown a strong association between GDF15 levels and age, although results of associations between GDF15 levels and gender, adiposity, and smoking status have been controversial [11, 13, 14, 29–36]. Our data showed higher GDF15 levels in males and current smokers but not in obese subjects. This probably reflects the heterogeneity and broad spectrum of patients enrolled in different studies, artifacts of a small sample size, and gene-environmental interactions.

### 4.3. Metabolic Factors and GDF15 Levels

We observed a strong association between higher GDF15 concentrations and cardiometabolic risk factors, including hypertension, diabetes, smoking, and metabolic syndrome which precede the onset of overt cardiovascular diseases. Several studies have reported significantly lower TCHO and LDLC levels with increased GDF15 levels [6, 16, 37], which is inconsistent with our findings. Our study population was relatively young compared to that of other studies; however, it is unknown whether differences in ethnicity and age may have caused the difference. Vila et al. reported increased insulin resistance with increasing GDF15 levels [14], and we also found a trend of increased HOMA-IR with increasing GDF15 levels. Higher levels of fasting plasma glucose and elevated blood pressure have also been noted in other populations as well as in ours [29–36]; however, our data showed that the association was predominantly age related.

### 4.4. Association with GDF15 Inflammatory Biomarkers and Oxidative Stress

Inflammation and oxidative stress provide potent stimuli for GDF15 production under pathological conditions [1, 17, 38–42]. Significant associations between GDF15 levels and CRP levels have previously been reported in patients with non-ST elevation acute coronary syndrome, diabetes, morbid obesity, and cancer [29, 30, 32, 33, 43]. Our results also showed a similar trend in a relatively healthy population. Eggers et al. analyzed 1004 elderly German community dwellers and found an independent association between GDF15 and biomarkers of endothelial activation, both E- and P-selectins, sVCAM1, and sICAM1 [44]. These adhesion molecules are involved in the recruitment of leukocytes into the vessel wall, which is commonly regarded as a key step in the initiation of atherosclerosis. In a relatively young population, we further found a significant association between GDF15 and other inflammatory and oxidative stress markers including sTNFRII and homocysteine in our Taiwanese cohort. These results suggest that GDF15 may reflect endothelial activation and vascular inflammation and thus that multiple pathways are involved in the development and progression of atherosclerosis.

### 4.5. Association with GDF15 Levels and Adipokines

A recent study reported that GDF15 is expressed in adipose tissue both in humans and in mice and that it is a secretory product of adipocytes both in pre- and in differentiated adipocytes [17]. Furthermore, GDF15 mRNA levels were positively correlated with adiponectin mRNA, and recombinant GDF15 increased adiponectin secretion by differentiated human adipocytes [17]. GDF15 transgenic mice have also been found to have less white adipose tissue and a reduced inflammatory response [18]. We found significant associations between LCN2 levels and GDF15 in this study, supporting that GDF15 is a novel adipokine which may play a paracrine role in the modulation of adipose tissue function.

### 4.6. Association with GDF15 Levels and Renal Function

Circulating GDF15 levels have been shown to be highly significantly associated with renal function such as serum creatinine, eGFR, and markers of acute renal injury including cystatin C and LCN2 [29–36]. Although the association of GDF15 with renal function has suspect validity when a Bonferroni correction is stringently applied in multiple tests, our data still showed a strong association between GDF15 levels and LCN2. These results suggest that GDF15 is highly correlated with acute and chronic renal dysfunction, which is also a risk factor for future cardiovascular events.

### 4.7. Association between GDF15 Levels and All-Cause Mortality in Patients with PAD and CAD

The serum level of GDF15 has been increasingly reported to be a powerful predictor of all-cause mortality in healthy subjects and in those with diseases [5–10]. We further extended this observation to patients with high-risk PAD and those with angiographically documented CAD. Of note, the prognostic information provided by GDF15 level was better than that provided by CRP, which has been shown to be a potent predictor of the risk of short- and long-term mortality [45]. However, the precise biological roles of GDF15 in the association with adverse outcomes are still poorly understood. Previous studies of genetically engineered animals have shown that GDF15 appears to provide protection against cardiac injury via anti-inflammatory [46], antiapoptotic [41], and antihypertrophic [47] pathways. These apparently conflicting findings could be explained by a biphasic effect of GDF15 observed on progression of cancer: inhibition of carcinogenesis in normal tissue at early stages of tumor development and promotion of tumor at late stages of the disease [48]. Another possibility is that elevated GDF15 level is induced by upstream proinflammatory cytokines and represents an endogenous protective effort trying to limit the cardiovascular damage [49]. Therefore, whether *GDF15* genetic polymorphisms can predict mortality would be of interest. Our study suggests that the influence of baseline GDF15 levels due only to GDF15 polymorphisms may not be large enough to alter the risk of mortality risk in patients with PAD or CAD. As a key secretory cytokine in response to multiple cellular stressors, GDF15 is nonspecific and parallels the elevation of other risk markers. In addition, a lot of PAD-/CAD-associated comorbidities have been demonstrated to affect circulating GDF15 levels. Thus, the observed association between GDF15 levels and mortality in PAD/CAD patients is probably independent of genetic influences.

### 4.8. Limitations

There are several limitations to this study. First, the genetic association study did not include any functional analysis. Second, the sample size of PAD patients is relatively small with inadequate power to exclude associations between GDF15 genetic variants and all-cause mortality. In addition, only association and not causation can be inferred from the results of our cohort studies. A larger sample size with a Mendelian randomization approach may help to elucidate whether a causal association exists between GDF15 and all-cause mortality. Third, we did not perform multivariate analysis to determine whether GDF15 level is an independent predictor for all-cause mortality, because the number of cases of mortality was too small to obtain a reliable estimate of *β*-coefficients from the multivariate Cox proportional hazard regression model. Finally, the examined subjects were ethnically Chinese, and hence, caution should be exercised when extrapolating our results to other ethnic groups.

## 5. Conclusion

Both genetic and nongenetic factors, including cardiometabolic inflammatory markers, oxidative stress, and adipokines are strongly associated with GDF15 levels. As a key secretory cytokine in response to multiple cellular stressors, GDF15 serves as a prognostic predictor of all-cause mortality in diverse human disorders including high-risk PAD and angiographically documented CAD. Further investigations regarding the signaling pathways of GDF15 may help to discover novel therapies against PAD and CAD complications.

## Supplementary Material

Supplementary Table 1. Seven single nucleotide polymorphisms (SNPs) near and at the GDF15 in this study. Supplementary Figure 1. Linkage disequilibrium (LD) observed across SNPs of GDF15. The color scale in each square indicates the level of LD between the SNP pair. Supplementary References

## Figures and Tables

**Table 1:** Baseline characteristics of the study subjects from the healthy cohort.

	Subjects
Number	612
Age (years)	46.2 ± 10.0
Systolic BP (mmHg)	113.1 ± 16.1
Diastolic BP (mmHg)	75.0 ± 10.0
Mean BP (mmHg)	87.7 ± 11.2
Total cholesterol (mg/dL)	198.8 ± 36.4
HDL cholesterol (mg/dL)	53.0 (45.0–65.0)
LDL cholesterol (mg/dL)	116.1 ± 32.9
Triglyceride (mg/dL)	115.0 (76.0–165.8)
Body mass index (kg/m^2^)	24.3 ± 3.5
Diabetes mellitus (%)	5.1
Current smokers (%)	19.3
Fasting plasma glucose (mg/dL)	93.0 (88.0–99.0)
Fasting serum insulin (*μ*U/mL)	7.97 (6.09–10.97)
HOMA-IR index	1.86 (1.40–2.61)
GDF15 (pg/mL)	535.0 (415.3–716.5)
Adiponectin (mg/L)	6.0 (3.7–9.2)
Leptin (ng/mL)	14.90 (8.15–25.95)
Resistin (ng/mL)	14.8 (10.3–22.6)
Lipocalin 2 (ng/mL)	71.8 (53.2–94.1)
CRP (mg/L)	0.63 (0.27–1.34)
Fibrinogen (mg/dL)	264.9 ± 70.3
sE-selectin (ng/mL)	50.4 (36.0–65.9)
sP-selectin (ng/mL)	94.7 (65.7–169.2)
SAA (*μ*g/mL)	3.6 (1.7–6.2)
sICAM1 (ng/mL)	231.1 (180.8–278.2)
sVCAM1 (ng/mL)	479.0 (409.0–549.0)
MMP1 (pg/mL)	188.1 (100.4–399.1)
MMP2 (ng/mL)	121.9 (102.7–140.1)
MMP9 (ng/mL)	112.5 (75.6–169.1)
MCP1 (pg/mL)	59.8 (42.7–82.7)
sTNFRII (pg/mL)	3107.3 (2653.6–3740.0)
Homocysteine (mg/L)	9.6 (7.9–11.5)
Creatinine (mg/dL)	1.0 (0.8–1.1)
eGFR (mL/min/1.73 m^2^)	81.03 ± 14.94
Urinary ACR (mg/g)	4.69 (3.18–8.94)
Urinary 8-OHdG (ng/mg Cr)	33.3 (24.7–44.6)

Data are presented as mean ± SD, percentage, or median (interquartile range) as appropriate. BP: blood pressure; HDL: high-density lipoprotein; LDL: low-density lipoprotein; HOMA-IR index: homeostasis model assessment of insulin resistance index; CRP: C-reactive protein; sE-selectin: soluble E-selectin; sP-selectin: soluble P-selectin; SAA: serum amyloid A; sICAM1: soluble intercellular adhesive molecule 1; sVCAM1: soluble vascular cell adhesive molecule 1; MMP1: matrix metalloproteinase 1; MMP2: matrix metalloproteinase 2; MMP9: matrix metalloproteinase 9; MCP1: monocyte chemotactic protein 1; sTNFRII: soluble tumor necrosis factor-alpha receptor 2; eGFR: estimated glomerular filtration rate; ACR: albumin-to-creatinine ratio; 8-OHdG: 8-hydroxy-2′-deoxyguanosine-to-urinary creatinine ratio.

**Table 2:** Association of circulating growth differentiation factor 15 (*GDF15*) locus genotypes with GDF15 level in subjects from the healthy cohort.

SNP number	Minor allele	MAF	Model	Genotypes	GDF15 levels Median [interquartile range] pg/mL (*N*)	*P* value	FDR *P* value
rs749451	C	0.482	Additive	CC	556.0 [414.0–808.0] (135)	0.088	0.088
				CT	541.5 [434.0–685.0] (292)		
				TT	513.0 [382.0–712.5] (156)		
			Recessive	TT + CT	531.0 [415.5–687.5] (448)	0.396	0.396
				CC	556.0 [414.0–808.0] (135)		
			Dominant	TT	513.0 [382.0–712.5] (156)	0.056	0.098
				CT + CC	546.0 [426.0–719.5] (427)		

rs888663	G	0.167	Additive	GG	428.0 [339.0–634.0] (18)	0.052	0.060
				GT	529.0 [414.0–687.5] (159)		
				TT	543.5 [420.0–727.0] (406)		
			Recessive	TT + GT	538.0 [419.0–715.0] (565)	0.096	0.114
				GG	428.0 [339.0–634.0] (18)		
			Dominant	TT	543.5 [420.0–727.0] (406)	0.103	0.144
				GT + GG	520.0 [398.0–685.0] (177)		


rs8101804	T	0.318	Additive	CC	509.0 [406.0–661.0] (278)	0.008	0.019
				CT	554.0 [415.5–736.5] (239)		
				TT	602.0 [434.0–907.0] (65)		
			Recessive	CC + CT	532.0 [413.0–699.0] (517)	0.098	0.114
				TT	602.0 [434.0–907.0] (65)		
			Dominant	CC	509.0 [406.0–661.0] (278)	0.012	0.027
				CT + TT	561.5 [419.0–786.5] (304)		

rs1227731	A	0.164	Additive	AA	814.5 [531.0–937.5] (20)	9.51 × 10^−8^	4.87 × 10^−7^
				AG	608.0 [457.0–828.0] (149)		
				GG	513.0 [401.0–653.5] (411)		
			Recessive	GG + AG	532.5 [413.5–703.0] (560)	0.011	0.020
				AA	814.5 [531.0–937.5] (20)		
			Dominant	GG	513.0 [401.0–653.5] (411)	1.31 × 10^−7^	7.59 × 10^−7^
				AA + AG	637.0 [465.0–875.0] (169)		

rs1058587	G	0.281	Additive	CC	540.0 [411.5–730.5] (299)	0.049	0.060
				GC	546.0 [423.0–706.0] (237)		
				GG	462.0 [380.0–596.0] (45)		
			Recessive	CC + GC	542.0 [418.5–719.5] (536)	0.008	0.020
				GG	462.0 [380.0–596.0] (45)		
			Dominant	CC	540.0 [411.5–730.5] (299)	0.280	0.318
				GC + GG	531.0 [416.0–685.0] (282)		

rs1054564	C	0.164	Additive	CC	814.5 [531.0–937.5] (20)	1.39 × 10^−7^	4.87 × 10^−7^
				CG	606.5 [450.0–828.0] (150)		
				GG	511.5 [401.0–653.5] (412)		
			Recessive	GG + CG	532.0 [413.0–702.0] (562)	0.011	0.020
				CC	814.5 [531.0–937.5] (20)		
			Dominant	GG	511.5 [401.0–653.5] (412)	2.17 × 10^−7^	7.59 × 10^−7^
				CC + CG	633.0 [462.0–875.0] (170)		

rs16982345	A	0.280	Additive	AA	459.5 [378.0–596.0] (46)	0.047	0.060
				AG	547.5 [426.0–710.0] (234)		
				GG	539.5 [409.0–731.0] (302)		
			Recessive	GG + AG	543.5 [419.5–723.5] (536)	0.005	0.020
				AA	459.5 [378.0–596.0] (46)		
			Dominant	GG	539.5 [409.0–731.0] (302)	0.318	0.318
				AG + AA	532.5 [420.5–686.5] (280)		

N: number of subjects; MAF: minor allele frequency; FDR: false discovery rate. *P* value was adjusted for age, sex, body mass index, and current smoker.

**Table 3:** Association of growth differentiation factor 15 (*GDF15*) locus haplotypes with circulating GDF15 level in subjects from the healthy cohort.

Circulating GDF15 level					
	Haplotype	Frequency	Coefficient	*P* value	FDR *P* value
H1	TTCGCGG	36.81%	−0.0223	0.3118	0.37416
H2	CTCGGGA	27.07%	−0.0419	0.0846	0.1692
H3	CTTACCG	15.70%	0.1433	3.68 × 10^−7^	2.21 × 10^−6^
H4	TGTGCGG	13.44%	−0.0452	0.1484	0.2226
H5	CTCGCGG	2.87%	0.0055	0.9365	0.9365
H6	CGTGCGG	1.88%	−0.1642	0.0511	0.1533

SNP1: rs749451; SNP2: rs888663; SNP3: rs8101804; SNP4: rs1227731; SNP5: rs1058587; SNP6: rs1054564; SNP7: rs16982345; FDR: false discovery rate. Coefficients and *P* values were estimated based on haplotype trend regression analysis implemented in the HelixTree program. The selected haplotype was compared to all unselected haplotypes; *P* value was adjusted for age, sex, body mass index, and current smoker.

**Table 4:** Association between circulating growth differentiation factor 15 (GDF15) levels and measurable cardiovascular risk factors in subjects from the healthy cohort.

Clinical biochemical parameters^		Unadjusted		Adjusted for age and sex	
		*P*	*P* value	*P*	*P* value
Anthropology	Age	0.475	<0.001		
	Body mass index	0.087	0.035	0.017	0.681

Blood pressure^∗^	Mean BP	0.229	<0.001	0.096	0.028

Glucose metabolism^∗∗^	Fasting plasma glucose	0.074	0.075	−0.021	0.607

Lipid profiles^#^	HOMA-IR index	0.043	0.297	0.031	0.458
	Total cholesterol	0.082	0.049	0.006	0.885
	Triglyceride	0.079	0.057	0.023	0.583

Renal function	Creatinine	0.194	<0.001	0.129	0.004

Inflammation marker	CRP	0.126	0.002	0.082	0.049
	Fibrinogen	0.159	<0.001	0.096	0.020
	sE-selectin	0.167	<0.001	0.138	0.001^†^
	sP-selectin	0.014	0.726	−0.018	0.656
	sVCAM1	0.200	<0.001	0.128	0.002^†^
	sICAM1	0.151	<0.001	0.138	0.001^†^
	sTNFRII	0.211	<0.001	0.174	<0.001^†^
	MCP1	0.142	0.001	0.110	0.008
	MMP9	0.058	0.166	0.114	0.006

Adipokines	Leptin	−0.012	0.769	0.043	0.292
	Resistin	0.057	0.171	0.067	0.112
	Lipocalin 2	0.114	0.007	0.131	0.002^†^
	Adiponectin	0.033	0.420	0.032	0.433

Oxidative stress	Homocysteine	0.234	<0.001	0.190	<0.001^†^
	8-OHdG/creatinine	0.059	0.149	0.049	0.232

^^^Some variables in Table 1 were omitted from correlation analysis because of multicollinearity; ∗ were analyzed with the exclusion of subjects using antihypertensive drugs; ∗∗ were analyzed with the exclusion of subjects using hypoglycemic agent; # were analyzed with the exclusion of subjects using lipid-lowering agents; CRP: excluded subjects with CRP levels ≧ 10 mg/L. ^†^Statistically significant correlation after a Bonferroni correction.

**Table 5:** Circulating growth differentiation factor 15 (*GDF15*) levels according to the cardiovascular risk factors in subjects from the healthy cohort.

	(*N*)	Circulating GDF15 levels Median (interquartile range) pg/mL	*P* value
Sex^∗^	Male (323)	562.0 (419.5–749.5)	0.023
	Female (269)	513.0 (413.0–683.0)	
Current smoker^#^	No (475)	519.0 (417.0–701.5)	<0.001
	Yes (117)	589.0 (409.0–813.0)	
Hypertension	No (474)	506.5 (397.0–667.0)	0.002
	Yes (118)	688.0 (521.0–924.0)	
Diabetes mellitus	No (561)	529.0 (410.0–702.0)	<0.001
	Yes (31)	867.0 (539.5–1064.5)	
Obesity	No (355)	529.0 (407.5–702.5)	0.778
	Yes (237)	562.0 (422.0–730.0)	
Metabolic syndrome	No (481)	510.0 (400.0–679.0)	0.021
	Yes (111)	638.0 (507.5–872.5)	

*P* value was adjusted for age, sex, body mass index (BMI), and current smoker. ^∗^*P* value was adjusted for age, BMI, and current smoker. ^#^*P* value was adjusted for age, sex, and BMI. Obesity was defined as a BMI ≥ 25 kg/m^2^, according to the Asian criteria (WHO expert consultation, 2004). Metabolic syndrome was defined by the ATP III Asian criteria.

**Table 6:** Demographics of peripheral artery disease patients with or without mortality.

	Total (*n* = 86)	Survival (*n* = 76)	Mortality (*n* = 10)	*P* value
Age (years)	71.65 ± 10.90	71.89 ± 10.05	71.67 ± 16.00	0.951
Sex (male/female)	50/36	44/32	6/4	1.000
Body mass index (kg/m^2^)	23.94 ± 3.82	24.11 ± 3.69	23.46 ± 4.50	0.321
Smoking	33.3%	33.8%	30.0%	1.000
Diabetes mellitus	73.3%	75.0%	60.0%	0.447
Dyslipidemia	42.2%	43.8%	30.0%	0.507
Hypertension	84.9%	86.8%	70.0%	0.172
Congestive heart failure	17.4%	17.1%	20.0%	1.000
Stroke	15.1%	14.5%	20.0%	0.644
End-stage renal disease	41.2%	38.7%	60.0%	0.305
Coronary artery disease	55.8%	56.6%	50.0%	0.744
Rutherford grade 3: severe claudication	22.6%	24.3%	10%	
Rutherford grade ≧ 4: critical limb ischemia	77.4%	75.7%	90%	0.443
CRP level (mg/L)^#^	0.80 (0.26–2.12)	0.69 (0.23–1.91)	1.31 (0.69–2.19)	0.095^∗^
GDF15 level (pg/mL)	3448.7 (1713.5–5427.9)	2849.4 (1669.5–5320.7)	5749.6 (3530.7–7072.0)	0.028^∗^
rs1054564 C-allele carriers	30.2%	28.9%	40%	0.482

Data are presented as mean ± SD, number, percentage, or median (interquartile range) as appropriate. ^#^The CRP level data of 1 survivor and 1 nonsurvivor were missing. ^∗^Mann–Whitney *U* test was used to compare the two groups.

**Table 7:** Demographics of coronary artery disease patients with or without mortality.

	Total (*n* = 481)	Survival (*n* = 454)	Mortality (*n* = 27)	*P* value
Age (years)	65.6 ± 11.3	64.9 ± 11.0	77.1 ± 9.3	<0.001
Sex (male/female)	388/93	370/84	18/9	0.058
Body mass index (kg/m^2^)	26.0 ± 4.0	26.0 ± 4.0	25.2 ± 4.2	0.303
Smoking	24.3%	24.7%	18.5%	0.645
Diabetes mellitus	44.3%	43.2%	63.0%	0.044
Dyslipidemia	47.4%	48.5%	29.6%	0.057
Hypertension	78.2%	77.8%	85.2%	0.364
Congestive heart failure	6.4%	4.6%	37.0%	<0.001
Stroke	6.0%	5.7%	11.1%	0.217
Acute coronary syndrome	7.7%	5.7%	40.7%	<0.001
PCI during hospitalization	77.1%	78.0%	63.0%	0.071
CRP level (mg/L)^∗∗^	2.50 (1.30–4.30)	2.40 (1.20–4.10)	3.7 (2.20-21.20)	<0.001^#^
GDF15 (pg/mL)	945.0 (666.8–1552.4)	913.4 (656.4–1403.3)	2480.4 (1437.2–4278.7)	<0.001^#^
rs1054564 C-allele carriers	31.1%	31.4%	25.9%	0.550

Data are presented as mean ± SD, number, percentage, or median (interquartile range) as appropriate. PCI: percutaneous coronary intervention; ^∗∗^the CRP level data of 11 survivors and 1 nonsurvivor were missing; ^#^Mann–Whitney *U* test was used to compare the two groups.
