# Supplementary material for: Growth Differentiation Factor 15 May Predict Mortality of Peripheral and Coronary Artery Diseases and Correlate with Their Risk Factors
Source: Mediators Inflamm. 2017 Jul 17;2017:9398401. doi: 10.1155/2017/9398401 (PMC5535745; doi:10.1155/2017/9398401)
Supplement: Supplementary file 1 — Supplementary Table 1. Seven single nucleotide polymorphisms (SNPs) near and at the GDF15 in this study. Supplementary Figure 1. Linkage disequilibrium (LD) observed across SNPs of GDF15. The color scale in each square indicates the level of LD between the SNP pair. Supplementary References [file 9398401.f1.pdf]

Supplementary Table 1. Seven single nucleotide polymorphisms (SNPs) near and at the *GDF15* in this study

Gene	SNP number	Position	Location	Minor allele	MAF	HWE	Function	Primer sequence
PGPEP1	rs749451	18479647	3’-UTR	C	0.482	0.942		TaqMan SNP Genotyping Assays
PGPEP1	rs888663	18484922		G	0.167	0.979		TaqMan SNP Genotyping Assays
GDF15	rs8101804	18496107	nearGene-5 (-862)	T	0.318	0.672		TaqMan SNP Genotyping Assays
GDF15	rs1227731	18497903	intron	A	0.164	0.719		TaqMan SNP Genotyping Assays
GDF15	rs1058587	18499422	Exon2	G	0.281	0.912	Missense (H→D)	TaqMan SNP Genotyping Assays
GDF15	rs1054564	18499815	3’-UTR	C	0.164	0.736	MiRNASNP	TaqMan SNP Genotyping Assays
GDF15	rs16982345	18500722	nearGene-3’	A	0.28	0.967		TaqMan SNP Genotyping Assays

MAF：minor allele frequency；HWE：Hardy-Weinberg equilibrium

Supplementary Figure 1. Linkage disequilibrium (LD) observed across SNPs of GDF15. The color scale in each square indicates the level of LD between the SNP pair.

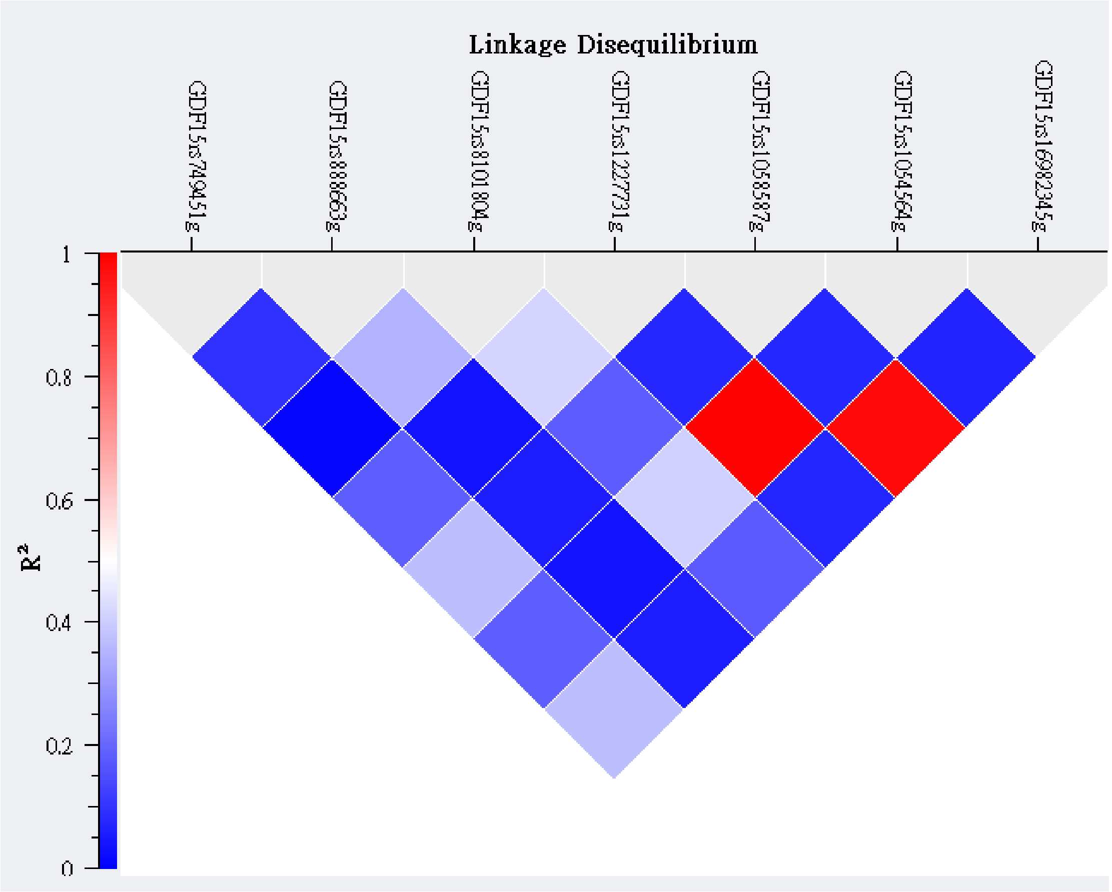

Supplementary References:

1. Chang PY, Wu TL, Tsao KC, et al. Microplate ELISAs for soluble VCAM-1 and ICAM-1. Ann Clin Lab Sci 2005; 35:312-7.
2. Tsao KC, Chang PY, Li CC, Wu TL, Sun CF, Wu JT. Development of a microplate ELISA for circulating E-selectin: assay characterization, comparison with a commercial kit, wand establishment of normal reference values. J Clin Lab Anal 2003; 17:97-101.
3. Wu TL, I Chen Tsai, Chang PY, et al. Establishment of an in-house ELISA and the reference range for serum amyloid A (SAA): complementarity between SAA and C-reactive protein as markers of inflammation. Clin Chim Acta 2007; 376:72-6.
4. Wu TL, Tsao KC, Chang CP, Li CN, Sun CF, Wu JT. Development of ELISA on microplate for serum C-reactive protein and establishment of age-dependent normal reference range. Clin Chim Acta 2002; 322:163-8.
5. Wu TL, Chang PY, Li CC, et al. Microplate ELISA for urine microalbumin: reference values and results in patients with type2 diabetes and cardiovascular disease. Ann Clin Lab Sci 2005; 35:149-54.
6. Chiou CC, Chang PY, Chan EC, et al. Urinary 8-hydroxydeoxyguanosine nd its analogs as DNA marker of oxidative stress: development of an ELISA and measurement in both bladder and prostate cancers. Clin Chim Acta 2003; 334:87-94.
